# Successful management of portal hypertension with splenomegaly and cavernous transformation of the portal vein: a rare case report

**DOI:** 10.1093/jscr/rjad607

**Published:** 2023-12-06

**Authors:** Simrah Sharjeel, Muhammad Abdullah

**Affiliations:** Medicine, Jinnah Sindh Medical University, Karachi, Pakistan; General Surgery, Indus Hospital and Health Network, Karachi, Pakistan

**Keywords:** extra hepatic portal venous obstruction, esophageal varices, spleno-renal shunt, non-cirrhotic portal hypertension, cavernous transformation, case report

## Abstract

Portal hypertension, often stemming from liver cirrhosis or vascular anomalies, can result in cavernous transformation of the portal vein, a rare condition associated with biliary obstruction, variceal hemorrhage, and splenomegaly. This case report details a unique occurrence of portal hypertension, splenomegaly, and cavernous transformation of the portal vein successfully managed through splenectomy and spleno-renal shunt. A 30-year-old female with a history of portal hypertension, portal gastropathy, and splenomegaly presented with left upper quadrant abdominal pain. She had previously undergone esophageal variceal ligation and required intermittent blood transfusions. Additional complications included easy bruising, heavy menstrual bleeding, and a prior episode of hematemesis. Clinical assessment confirmed splenomegaly, while a CT scan confirmed the diagnosis. A tailored surgical approach was chosen, leading to splenectomy and spleno-renal shunt.

## Introduction

Portal hypertension, a complex condition characterized by increased blood pressure within the portal venous system, often can result from liver cirrhosis, portal vein thrombosis, or vascular abnormalities [1]. One main consequence of portal hypertension is the cavernous transformation of the portal vein, a rare condition with an insidious presentation. It often manifests as biliary obstruction, variceal bleeding, and splenomegaly [2].

Herein, we report a rare case of portal hypertension, splenomegaly, and cavernous transformation of the portal vein treated with a splenectomy and spleno-renal shunt. This case report has been reported in line with the SCARE Criteria [3].

## Case presentation

A 30-year-old female with a history of portal hypertension, portal gastropathy, and splenomegaly presented to the outpatient department with pain and a dragging sensation in the left upper quadrant of her abdomen. She had previously undergone multiple sessions of endoscopic band ligation for esophageal varices and occasionally required transfusions for red blood cells and platelets. The patient also reported easy bruising and heavy menstrual bleeding. She had a history of melena and a single episode of hematemesis in the past. Notably, she had umbilical sepsis in infancy, which may have been a risk factor for her current diagnosis. Apart from this, her family history, drug history, and psychosocial history was unremarkable.

On examination, she appeared pale and lethargic but was alert and oriented. Abdominal examination revealed an enlarged and tense spleen extending 19 cm beyond the costal margin, while the liver was non-tender and palpable. The percussion note was tympanic, with no signs of fluid accumulation. Cardiovascular and respiratory exams were unremarkable.

**Figure 1:**
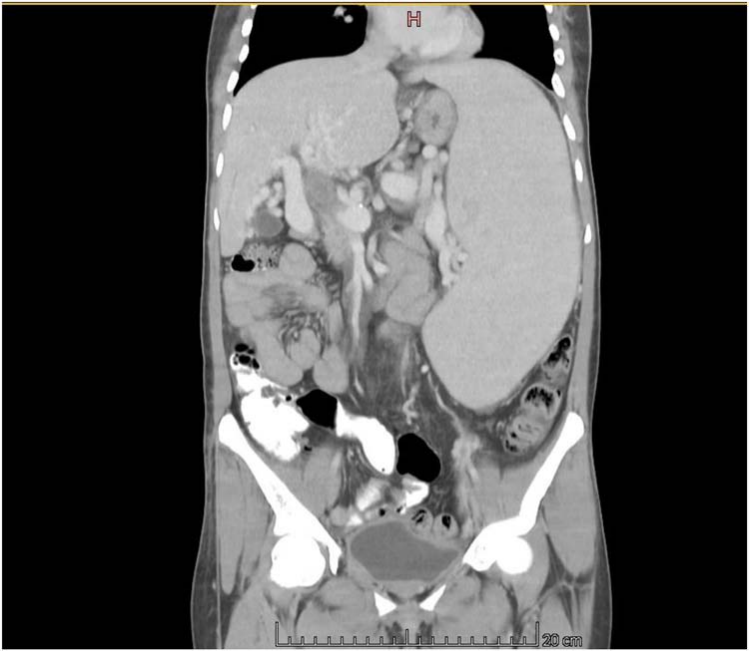
CT scan coronal view showing gross hepatosplenomegaly with large collaterals suggestive of portal venous hypertension with portal biliopathy. Chronic thrombus seen in SMV and portal confluence.

**Figure 2:**
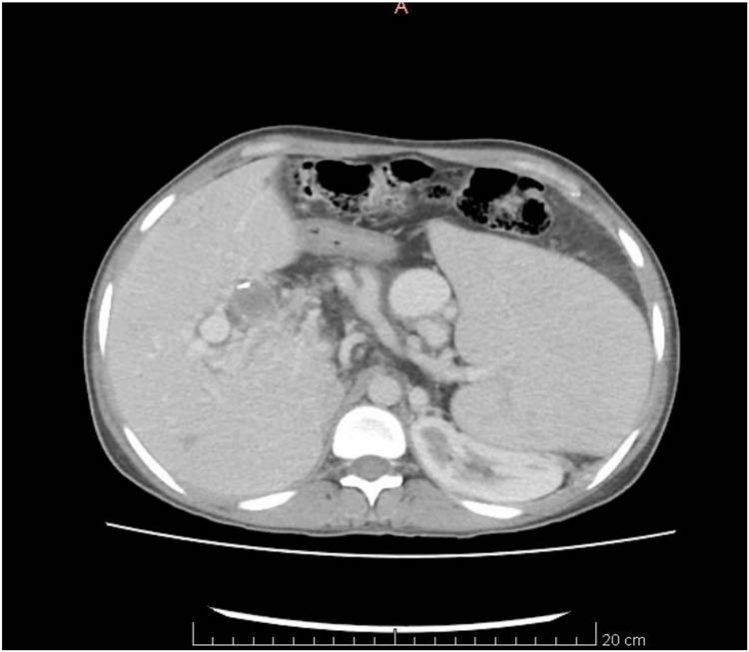
CT scan axial view.

The CT scan exhibited features consistent with portal hypertension and associated changes in the pre-portal venous system, liver, and spleen (Figs 1 and 2).

**Figure 3:**
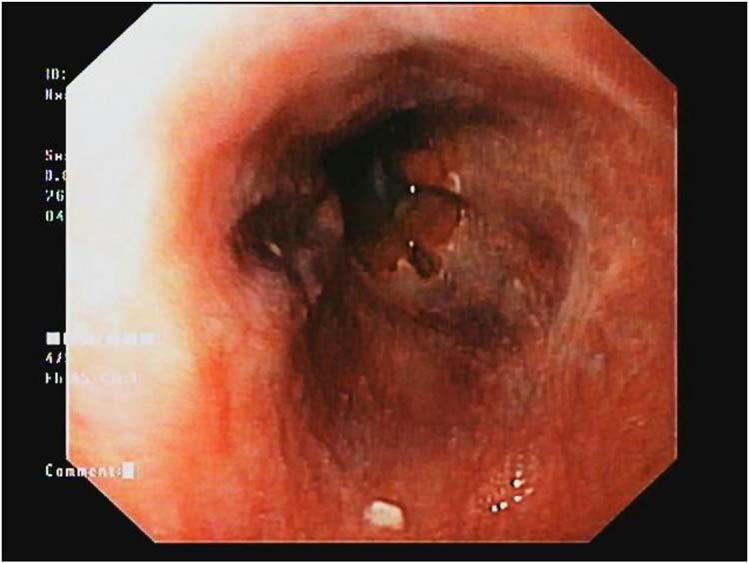
Upper GI scopes showing portal hypertension gastropathy.

The patient’s complete blood count indicated pancytopenia, while liver function tests showed no significant abnormalities. Upper gastrointestinal endoscopy showed the presence of small esophageal varices and severe portal hypertensive gastropathy (Fig. 3).

Based on the patient’s clinical history, examination findings, and imaging studies, the working diagnosis of cavernous transformation of the portal vein (complicated by portal hypertension with splenomegaly, portal gastropathy, and suspected cirrhosis) was decided upon. The patient was informed about her condition and consented to a high-risk surgical procedure involving splenectomy and spleno-renal shunt. She underwent pre-splenectomy vaccination.

The surgical procedure involved a Chevron incision. During the operation, an enlarged and firm spleen was encountered. Multiple dilated and tortuous varices were found around the spleen involving the splenocolic, phrenicocolic, and gastrocolic ligaments. The splenic vein was also dilated and tortuous. Splenic vein dissection was affected proximal to the splenic hilum to preserve maximum length for the subsequent shunt. Following removal of the spleen, an end-to-side, central (non-selective) spleno-renal shunt was performed with polydioxanone 5/0.

**Figure 4:**
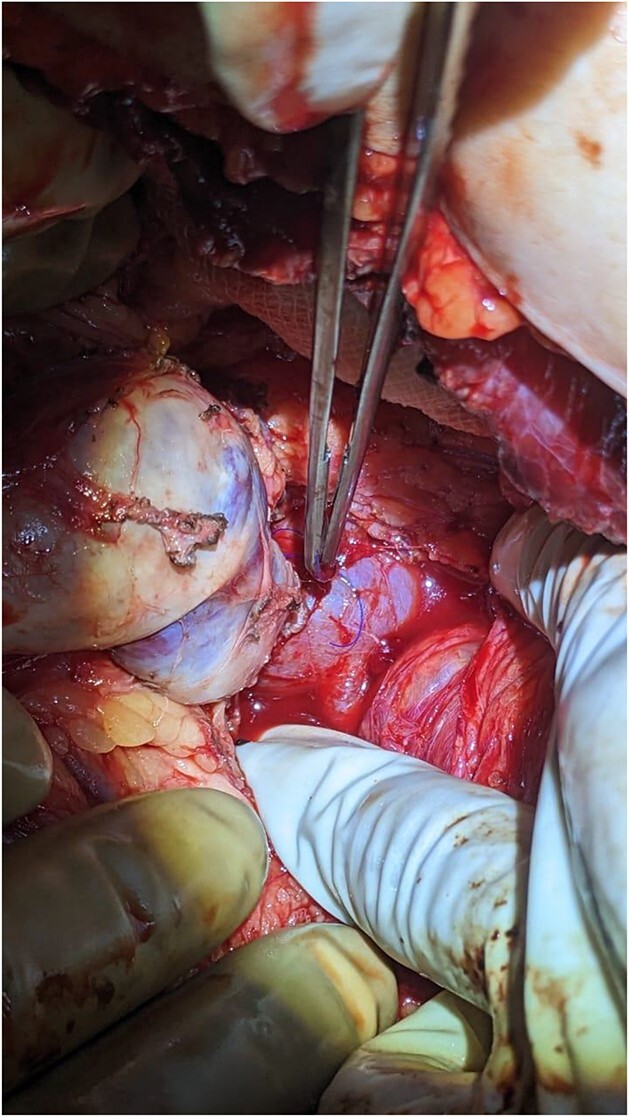
Proximal side-to-side spleno-renal shunt.

The patient was monitored for vital signs, pain, and fluid balance during the immediate post-operative period. She had an uneventful recovery and was discharged on the third day after surgery (Fig. 4).

Post-operative follow-ups were conducted two and four weeks after surgery. She has remained well and stable. Her quality of life has improved significantly, as has her functional class. Hematological tests done reveal a reversal of counts to normal levels. A repeated upper GI scope performed after a month showed complete resolution of gastric varices. She has been taking meat regularly, but there are no alarming signs of encephalopathy. She has been kept on regular surveillance at 3-month intervals.

## Discussion

Extra Hepatic Portal Vein Obstruction (EHPVO) is a rare condition with an uncertain prevalence. In India, it affects 0.84–13.6 individuals per 100 000, while in Western countries, portal venous thrombosis accounts for two to five percent of all variceal bleeds. EHPVO predominantly affects young adults, primarily between the ages of 10 and 30, and men are more commonly affected, with a male-to-female ratio of 2:1 [4]. Therefore, there is limited knowledge about risk factors, natural progression, and the long-term outcomes of surgical interventions.

Historically, EHPVO has been treated with surgical shunts, the largest series of which was conducted by Orloff *et al.*, who conducted two randomized controlled trials on emergency portocaval shunts. Their findings indicated that this procedure effectively stopped variceal bleeding with almost no chance of re-occlusion [5].

Predisposing factors for EHPVO include intra-abdominal infections, peritonitis, hypercoagulability, and portal venous stenosis or atresia. EHPVO and non-cirrhotic portal fibrosis are the leading causes of non-cirrhotic portal hypertension [6]. It is essential to consider our patient’s history of neonatal omphalitis, which may have contributed to the development of portal hypertension in this case. The infection spread in such instances may lead to portal pyemia, resulting in irreversible venous wall damage, either through thrombogenic predisposition, fibrotic narrowing, or both [7].

Due to its vast range of clinical manifestations, early EHPVO diagnosis is challenging. Common symptoms include abdominal pain, splenomegaly, recurrent variceal bleeding, and ascites. Splenomegaly causing hypersplenism is an important finding seen in patients with EHPVO. Even Though medical and endoscopic management is recommended for EHPVO, shunt surgery is indicated in rare cases such as portal biliopathy, hypersplenism, massive splenomegaly affecting the quality of life, growth retardation, and rare blood group [4].

Our patient had been undergoing endoscopic band ligation due to esophageal variceal bleeding. Although endoscopic management has temporarily alleviated variceal bleeding, it is not treating the underlying cause, making it unsuitable as a long-term treatment. Despite potential hazards due to collaterals, direct surgical procedures on the biliary system are often performed for EHPVO, providing a one-time solution for young patients with long life expectancies with almost no recurrence changes [8].

Therefore, our patient underwent a splenectomy and an end-to-side proximal spleno-renal anastomosis. The patient remained stable during follow-ups, and GI scope showed no changes related to portal hypertension. Overall, she showed signs of improvement at every flow-up.

## Conclusion

This case report highlights the importance of a comprehensive approach to managing Noncirrotic portal hypertension, especially concurrent with splenomegaly and recurrent variceal bleeding. Although surgical interventions such as a splenectomy and a spleno-renal shunt can improve outcomes such as seen with this case, due to the rarity of this condition, data on it is scanty.

Treatment plans tailored to each and close follow-up are necessary to achieve positive long-term outcomes and prevent complications. Further research and larger studies are needed to evaluate these surgical procedures’ long-term effectiveness and safety in patients.

## Data Availability

All data related to the content of this case report is present within the body of the manuscript.

## References

[1] Iwakiri Y, Trebicka J. Portal hypertension in cirrhosis: pathophysiological mechanisms and therapy. JHEP Rep 2021;3:100316. 10.1016/j.jhepr.2021.100316. 34337369 PMC8318926

[2] Wei B, Huang Z, Tang C. Optimal treatment for patients with cavernous transformation of the portal vein. Front Med (Lausanne) 2022;9:853138. 10.3389/fmed.2022.853138. 35402447 PMC8987530

[3] Agha RA, Franchi T, Sohrabi C. et al. The SCARE Guideline: updating consensus surgical CAse REport (SCARE) guidelines. Int J Surg 2020;84:226–30. 33181358 10.1016/j.ijsu.2020.10.034

[4] Wani ZA, Bhat RA, Bhadoria AS, Maiwall R. Extrahepatic portal vein obstruction and portal vein thrombosis in special situations: need for a new classification. Saudi J Gastroenterol 2015;21:129–38. 26021771 10.4103/1319-3767.157550 PMC4455142

[5] Orloff MJ . Fifty-three years' experience with randomized clinical trials of emergency portacaval shunt for bleeding esophageal varices in cirrhosis: 1958–2011. JAMA Surg 2014;149:155–69. Erratum in: *JAMA Surg*. 2014;**149**:543. 24402314 10.1001/jamasurg.2013.4045

[6] Gioia S, Nardelli S, Ridola L, Riggio O. Causes and management of non-cirrhotic portal hypertension. Curr Gastroenterol Rep 2020;22:56. 32940785 10.1007/s11894-020-00792-0 PMC7498444

[7] Prabhu R, Natarajan A, Krishna S, Thangavelu S. Portal pyaemia secondary to open haemorrhoidectomy: need for prophylactic broad spectrum antibiotics. BMJ Case Rep 2013;2013:bcr2013200222. 10.1136/bcr-2013-200222 PMC3703088 23814220

[8] Agarwal AK, Sharma D, Singh S. et al. Portal biliopathy: a study of 39 surgically treated patients. HPB (Oxford) 2011;13:33–9. 21159101 10.1111/j.1477-2574.2010.00232.x PMC3019539

